# Supplementary figures and images for: Polycomb repressive complex 1.1 coordinates homeostatic and emergency myelopoiesis
Source: eLife. 2023 Jun 2;12:e83004. doi: 10.7554/eLife.83004 (PMC10287155; doi:10.7554/eLife.83004)

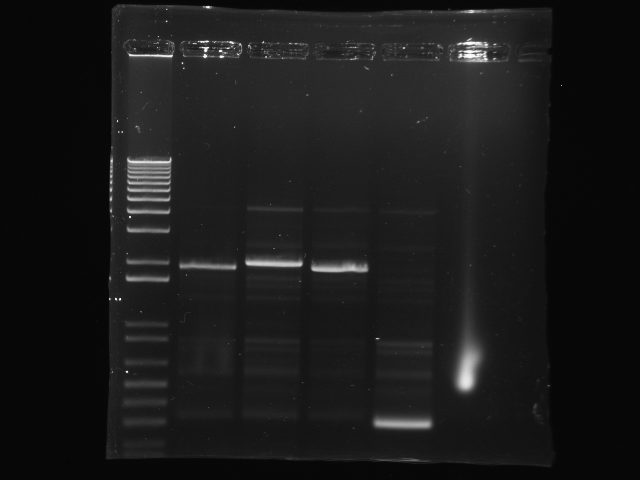

Supplement: Figure 1—figure supplement 1—source data 1. [file elife-83004-fig1-figsupp1-data1.zip › Figure 1-figure supplement 1-source data 1/Nakajima-Takagi et al, Figure 1ΓÇôfigure supplement 1C source data.tif]

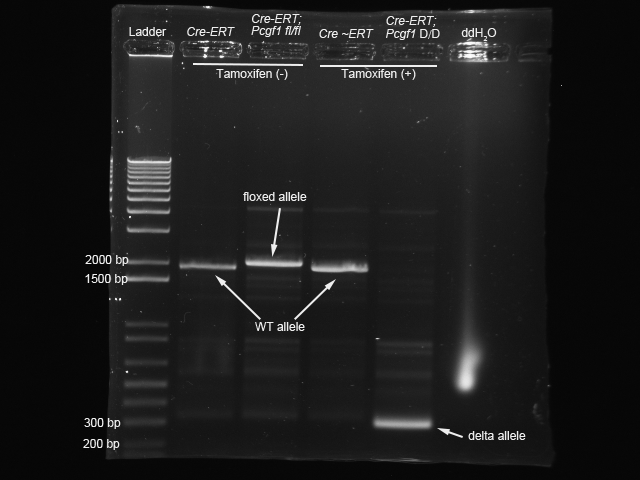

Supplement: Figure 1—figure supplement 1—source data 1. [file elife-83004-fig1-figsupp1-data1.zip › Figure 1-figure supplement 1-source data 1/Nakajima-Takagi et al, Figure 1ΓÇôfigure supplement 1C source data_annotated.tif]

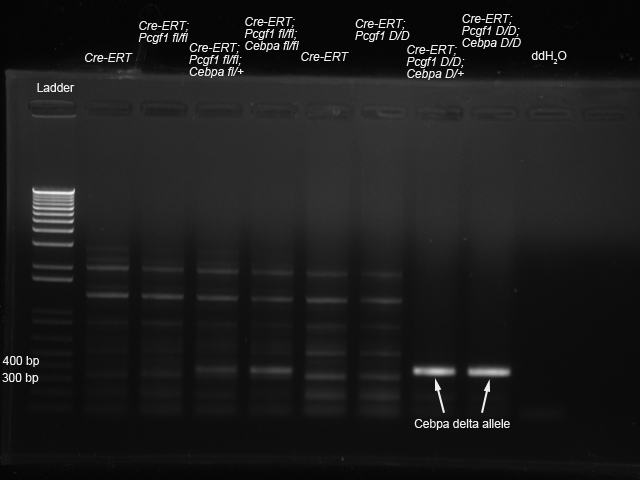

Supplement: Figure 2—figure supplement 1—source data 1. [file elife-83004-fig2-figsupp1-data1.zip › Figure 2-figure supplement 1-source data 1/Nakajima-Takagi et al, Figure 2ΓÇôfigure supplement 1A_source data 3_annotated.tif]

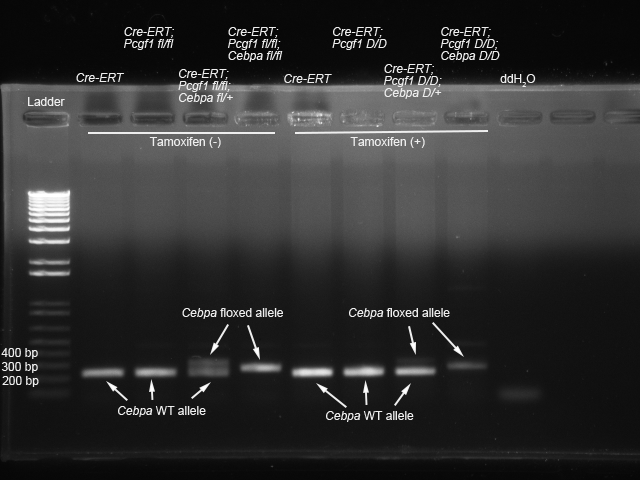

Supplement: Figure 2—figure supplement 1—source data 1. [file elife-83004-fig2-figsupp1-data1.zip › Figure 2-figure supplement 1-source data 1/Nakajima-Takagi et al, Figure 2ΓÇôfigure supplement 1A_source data 2_annotated.tif]

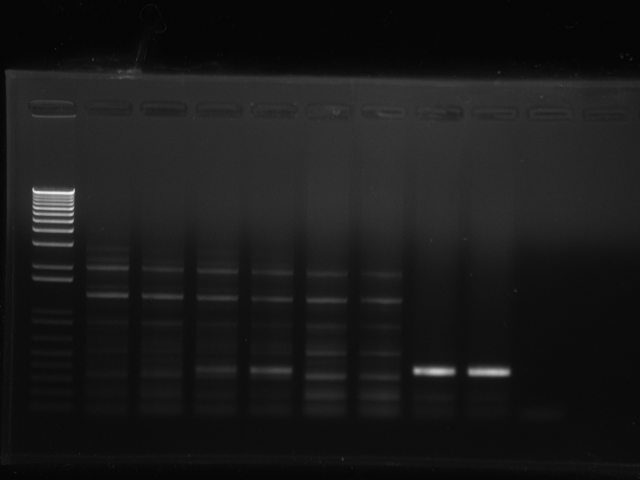

Supplement: Figure 2—figure supplement 1—source data 1. [file elife-83004-fig2-figsupp1-data1.zip › Figure 2-figure supplement 1-source data 1/Nakajima-Takagi et al, Figure 2ΓÇôfigure supplement 1A_source data 3.tif]

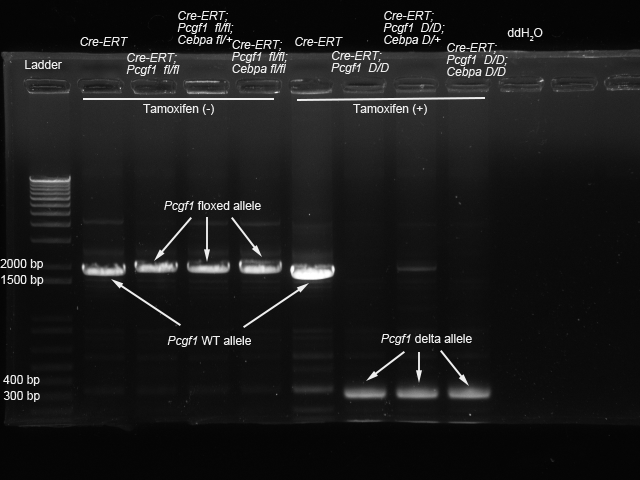

Supplement: Figure 2—figure supplement 1—source data 1. [file elife-83004-fig2-figsupp1-data1.zip › Figure 2-figure supplement 1-source data 1/Nakajima-Takagi et al, Figure 2ΓÇôfigure supplement 1A_source data 1_annotated.tif]

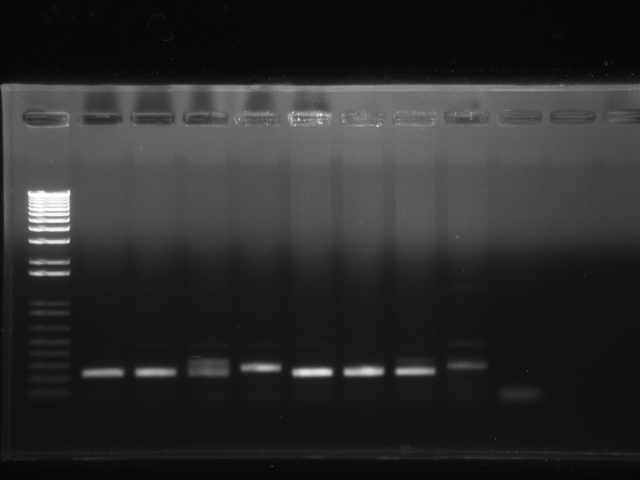

Supplement: Figure 2—figure supplement 1—source data 1. [file elife-83004-fig2-figsupp1-data1.zip › Figure 2-figure supplement 1-source data 1/Nakajima-Takagi et al, Figure 2ΓÇôfigure supplement 1A_source data 2.tif]

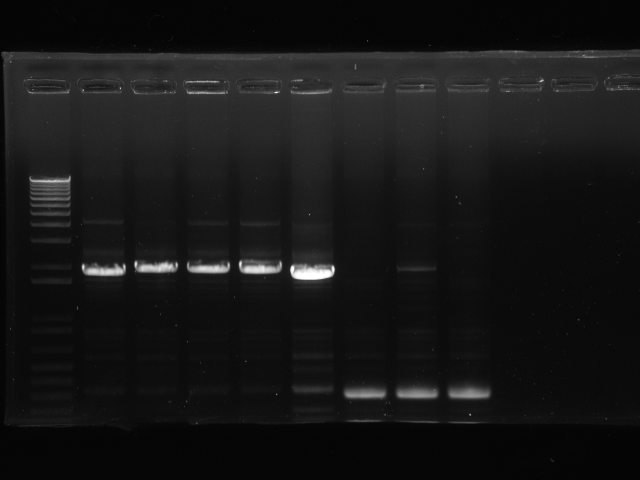

Supplement: Figure 2—figure supplement 1—source data 1. [file elife-83004-fig2-figsupp1-data1.zip › Figure 2-figure supplement 1-source data 1/Nakajima-Takagi et al, Figure 2ΓÇôfigure supplement 1A_source data 1.tif]
